# Lumen-Apposing Metal Stents for Endoscopic Transgastric Drainage of Pancreatic Fluid Collections in Children—A Case Report and Review of Safety and Efficacy

**DOI:** 10.3390/children12080965

**Published:** 2025-07-23

**Authors:** Irene Wen Hui Tu, Zong Jie Koh, Khek Yu Ho, Sivaramakrishnan Venkatesh Karthik, Vidyadhar Padmakar Mali

**Affiliations:** 1 Department of Paediatric Surgery, National University Hospital, Singapore 119074, Singapore; zong_jie_koh@nuhs.edu.sg (Z.J.K.); vidyadhar_mali@nuhs.edu.sg (V.P.M.); 2 Division of Gastroenterology, Department of Medicine, National University Hospital, Singapore 119074, Singapore; khek_yu_ho@nuhs.edu.sg; 3 Department of Paediatrics, National University Hospital, Singapore 119074, Singapore; venkatesh_karthik@nuhs.edu.sg

**Keywords:** lumen-apposing metal stent, paediatric, pancreatic fluid collection, walled-off necrosis, endoscopy, transgastric, necrectomy

## Abstract

**Background/Objectives:** Pancreatic fluid collections (PFCs) in acute pancreatitis require drainage when symptomatic or infected. Walled-off necrosis (WON) is difficult to drain with plastic stents alone. A lumen-apposing metal stent (LAMS) offers larger calibre drainage, lower migration risk than conventional methods, and the option of direct endoscopic necrosectomy through the stent. However, the paediatric literature on LAMSs is sparse. We report our institutional experience, and summarise current evidence on the feasibility, efficacy and safety of LAMSs for PFC drainage in children. **Methods:** We performed a retrospective study at the National University Hospital (NUH) and a full review of the literature on LAMS use in children for endoscopic trans-gastric drainage of PFCs from April 2012 to September 2024. **Results:** There were, respectively, 2 (males, 10 and 17 years) and 18 children who underwent endoscopic trans-gastric LAMS insertion for drainage of PFCs in acute pancreatitis in the NUH and across the nine included studies, which were published between 2015 and 2024. The technical and clinical success was 100%. There were no complications during insertion or indwell time (28 and 50 days in the NUH and 40 days, range of 7–100 days in the systematic review, respectively). Endoscopic removal of LAMSs was uneventful. There were no recurrent PFCs over a 4-month (1,7 months) and 12-month (range, 2–44 months) follow-up, respectively. Migration of LAMSs to colon following the collapse of the WON was reported in one case. **Conclusions**: An transgastric LAMS (with trans-stent necrosectomy) is a technically feasible method of drainage of WON following acute pancreatitis in children with minimal complications.

## 1. Introduction

The incidence of acute pancreatitis in children is increasing (3.6–13/100,000 children-year) [1,2]. Pancreatic fluid collections (PFCs) may occur in 23% to 61% of paediatric pancreatitis [1,3,4]. As per the North American Society for Paediatric Gastroenterology, Hepatology and Nutrition (NASPGHAN), a significant proportion of acute PFCs in children resolve spontaneously without requiring any drainage [5]. However, symptomatic or infected PFCs may require therapeutic drainage (18.2% of pseudocysts and 35.7% of walled-off necrosis (WON)) [1,5].

In children, NASPGHAN recommends endoscopic methods as favoured over open approaches, which aligns with adult guidelines for a step-up, minimally invasive approach [5,6,7,8]. The endoscopic management includes endoscopic transluminal drainage of PFCs and insertion of plastic stents with the option of direct endoscopic necrosectomies for clearance [6]. One systematic review in children showed 88.7% and 92.3% success after one and two interventions, respectively, with plastic stents used in the majority [9,10].

Issues with plastic and biliary fully-covered self-expanding metallic stents (FCSEMS), such as blockage and migration, have led to the development and use of lumen-apposing metal stents (LAMS) since 2012 [9,10,11,12,13]. Unlike traditional narrow-calibre plastic stents, LAMSs have been shown to be particularly resistant to occlusion by necrotic tissue within the pseudocyst in adults due to their larger lumen and the option of direct endoscopic necrosectomy via the stent itself [14].

However, the literature on endoscopic transgastric LAMSs for PFCs in paediatric pancreatitis is limited to case reports and small series, which precludes drawing firm conclusions. We report our experience with two cases and a systematic review of the literature on the feasibility, efficacy, and safety of LAMSs in the endoscopic trans-gastric drainage of PFCs following acute pancreatitis in children.

## 2. Materials and Methods

This is a retrospective study of the first 2 cases at the National University Hospital, Singapore, and a review of the literature on the use of LAMSs for endoscopic trans-gastric drainage of PFCs in paediatric acute pancreatitis. This study was approved by the Central Institutional Review Board (Ref: 2019/2040).

**Figure 1:**
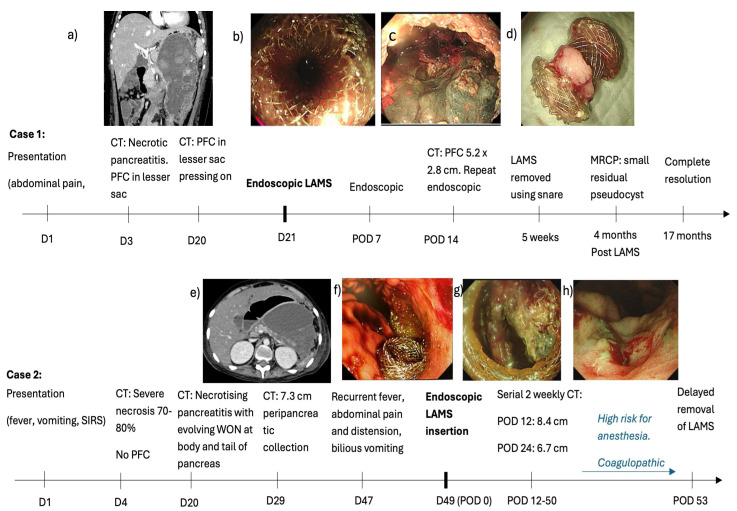
Clinical course of two cases of acute pancreatitis with pancreatic fluid collections (PFCs). (**a**) Day 20 CT scan showing a large PFC  abutting the stomach, causing a significant mass effect (**b**) Endoscopic ultrasound (EUS)-guided cysto-gastrostomy on Day 21 using a Boston Hot AXIOS LAMS (10 length × 15 mm diameter). (**c**) Necrosectomy repeated 14 days later (**d**) LAMS removed at 5 weeks (**e**) CT scan shows an enlarging PFC causing compression and anterior displacement of stomach. (**f**) LAMS deployed on day 49 of illness. (**g**) Endoscopic necrosectomy performed for persistent WON 50 days after LAMS insertion. (**h**) Patent healthy cystogastrostomy tract after removal of LAMS..

(1) A 10-year-old boy presented with a 1-day history of abdominal pain, vomiting and fever (37.7 Celsius). Amylase and lipase were elevated to more than 3 times normal (863 U/L and 969 U/L, respectively). A computerised tomography (CT) scan on day 3 revealed an oedematous pancreas with PFC in the lesser sac (6.6 × 6.0 cm). Oral feeds could be commenced and progressed to a full diet by day 16 of admission. Vomiting recurred together with high-spiking fevers by day 18. A CT scan on day 20 revealed preserved homogenous enhancement of the pancreas and a large PFC (10.7 × 9.9 × 19.1 cm) with internal debris and well-defined walls that were abutting the stomach, causing a significant mass effect (Figure 1a). On day 21, under general anaesthesia, an endoscopic ultrasound (EUS)-guided cysto-gastrostomy was performed and a Boston Hot AXIOS LAMS (10 length × 15 mm diameter) was inserted (Figure 1b). Endoscopy was repeated one week later to perform a necrosectomy (using a grasper, Roth net, and snare) and lavage using hydrogen peroxide. A CT scan at 2 weeks after LAMS insertion revealed a reduction in the size of the PFC to 5.2 × 2.8 cm. Necrosectomy was repeated for persistent PFC on day 14 after the initial insertion of the LAMS (Figure 1c). Antibiotics were tailored to the results of the culture of the necrotic tissue obtained at endoscopy. His fevers settled, and his general condition improved with the re-commencement and establishment of full feeds by week 5 of admission. The LAMS was removed endoscopically using a snare at 5 weeks after insertion (Figure 1d). He was discharged well at 2 months after admission. Magnetic resonance cholangiopancreatography 4 months after discharge showed no pancreatic duct disruption or any pancreatic or bile duct anomaly. He remains well at 24 months with complete resolution of the PFC on follow-up ultrasound scans.

(2) A 17-year-old boy with relapsed pre-B cell acute lymphocytic leukaemia and Klinefelter syndrome presented with fever, nausea, and vomiting 13 days after receiving Peg-asparaginase. Amylase (890 U/L) and lipase (2084 U/L) were elevated to more than 3 times the upper limit of normal. A CT scan revealed acute pancreatitis with no obvious necrosis or PFC. This progressed to severe necrosis of 70–80% of the pancreatic parenchyma on day 4. There was no significant PFC. CT scan on day 20 for new-onset fever (>38 C) revealed necrotising pancreatitis with evolving WON in the body and tail of the pancreas. He developed thrombocytopenia in addition to his deranged INR, resulting in a spontaneous retroperitoneal hematoma on day 29. The CT scan, which was performed for the retroperitoneal haematoma, revealed similar appearances of the pancreas. He developed bilious vomiting with abdominal pain and distension by day 47. A CT scan revealed an enlarging PFC (12.2 cm from 7.3 cm), resulting in compression and anterior displacement of the stomach (Figure 1e). There was internal debris suggestive of WON. On day 49, under general anaesthesia, he underwent EUS and endoscopic cyst-gastrostomy with deployment of LAMS like case 1 as above. (Figure 1f) The aspirated cyst fluid was rich in amylase (1724 U/L vs. serum amylase 31 U/L), and cyst fluid culture yielded Stenotrophomonas and Actinomyces. He developed pancreatic endocrine and exocrine insufficiency requiring long-term insulin for hyperglycemia and transient octreotide for loose stools, respectively. He developed other co-morbidities such as mycobacterium tuberculosis pneumonia requiring a 4-drug anti-tuberculosis regimen, cytomegalovirus viraemia requiring ganciclovir, left iliac vein thrombosis, hypertension due to chronic steroid use, and BK virus haemorrhagic cystitis, which responded to ciprofloxacin and herpes simplex keratitis in his right eye. Further endoscopy for necrosectomy could not be performed because of his poor general condition, high risk for anaesthesia, and coagulopathy. The LAMS was left in situ with surveillance CT scans, which revealed a progressive decrease in maximum WON size from 12.2 cm to 6.7 cm and 6.2 cm on days 63, 75, and 93 of illness and days 12, 24, and 50 of the LAMS, respectively. In view of the improving and stable general condition with persistent residual WON, endoscopic necrosectomy was performed as per case 1 on day 93 of illness and day 50 of LAMS insertion (Figure 1g). The necrosectomy seemed complete with healthy tissue within the cavity. Hence, the LAMS was removed using a snare. At the end of the procedure, a patent healthy cystogastrostomy tract could be visualised (Figure 1h). A CT scan on the day after the LAMS removal revealed no debris and a small residual PFC. Our plan was to monitor progression with surveillance scans. Unfortunately, the family decided to bring the boy back home overseas, where he succumbed after 3 weeks despite continued management.

### Literature Review

To perform as comprehensive a literature review as possible, we followed the PRISMA (Preferred Reporting Items for Systematic review and Meta-Analysis) 2020 checklist and searched the electronic databases of PubMed, Google Scholar, Science Direct, and EMBASE from April 2012 (with the first reported use of LAMSs for PFCs in the literature) until September 2024 using the following search terms: (“pancreatic fluid collection” OR “PFC” OR “walled-off necrosis” OR “WON” OR “pancreatic pseudocyst”) AND (“lumen apposing metal stent” OR “LAMS” OR “AXIOS stent”) AND (“pediatric” OR “paediatric” OR “children” OR “child” OR “adolescent” OR “infant” OR “teenager”) AND (“endoscopic drainage” OR “endoscopic ultrasound” OR “EUS-guided” OR “transgastric”) [10,15]. A database search was conducted from 5 September 2024 to 8 February 2025. References of identified studies were screened to identify further eligible studies. We included studies that (1) reported paediatric patients (<18 years) with (2) pancreatitis only (3) undergoing transgastric drainage using (4) LAMSs for PFCs, (5) provided sufficient clinical data, and (6) were in English. Titles and abstracts of all eligible studies retrieved were independently screened by two reviewers (TI and MV), with disagreements resolved by discussion. Full texts were assessed in detail for final inclusion in the review. Primary outcomes were technical and clinical success. Clinical success is defined as the resolution of PFCs, as verified on imaging or endoscopy. Secondary outcomes were procedure-related complications and recurrence of PFCs requiring further interventions. Studies were appraised using the Joanna Briggs Institute’s (JBI) critical appraisal checklist for cohort and case series/reports by 2 authors (TI, MV), with any discrepancies being resolved with a third author (ZJ) [16]. Statistical pooling was not performed due to heterogeneity and the descriptive nature of the included studies. As per PRISMA 2020 and Cochrane guidance, narrative synthesis is an acceptable approach when meta-analysis is not feasible [15,17].

## 3. Results

The National University Hospital is a tertiary academic medical centre in Singapore with adult and paediatric facilities at one site. The procedures were performed by a senior adult endoscopist with extensive expertise in paediatric interventional endoscopies. The time from presentation to insertion of LAMSs was 3 weeks and 7 weeks, respectively. There were no procedure-related or stent-related complications over a stent dwell time of 28 and 50 days, respectively. Stent removals were uneventful. There was no recurrence of PFCs in the one survivor.

**Figure 2:**
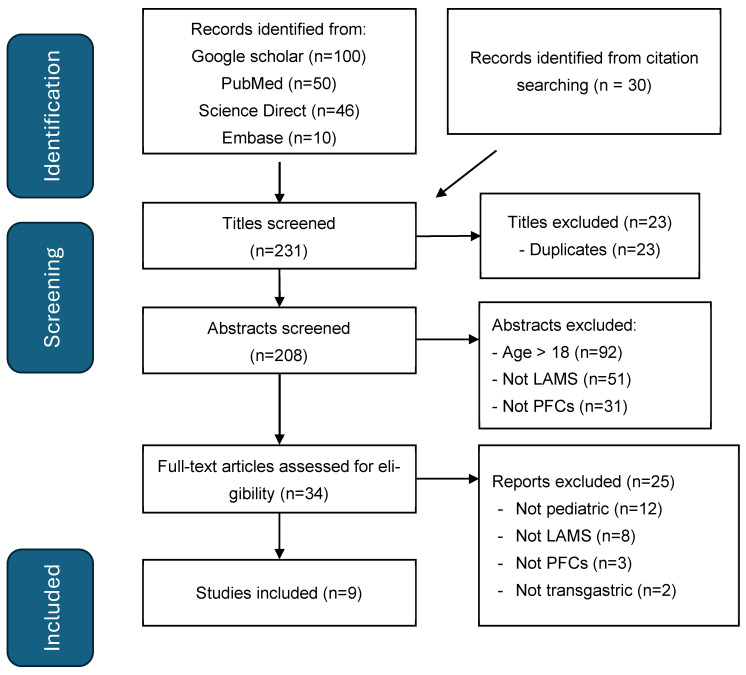
PRISMA flow diagram for LAMSs in pancreatitis in children.

Our literature search produced 231 records, of which 23 were duplicates. Through titles and abstract screening, a further 174 reports were excluded as per the following criteria: adult population (patients aged >18 years), reports of LAMS use for indications other than for drainage of PFCs, drainage conducted through the oesophagus, and reports of other methods of PFC drainage not attributed to LAMSs. The full text of the remaining 34 reports was reviewed and analysed. Only reports describing cases or case series of LAMS deployment for trans-gastric drainage of PFC (for both WON and pancreatic pseudocyst) in children ≤ 18 years were included. There were 9 studies that fulfilled the inclusion criteria after full-text review (Figure 2) (Table 1). All studies met the majority of the JBI checklist items; common limitations are in line with the nature of case reports and small case series, such as a lack of consecutive case inclusion. (Supplemental Material). There were two studies from the same author, one with single-institution and one with multi-institutional experience [18,19]. From a review of the two studies, we could identify five individual children who are included in this systematic review (Table 1).

### 3.1. Patient Characteristics

There were 18 children who underwent endoscopic trans-gastric LAMS insertion for drainage of PFC in acute pancreatitis across the nine included studies, which were published between 2015 and 2024 (Table 1, Table 2 and Table 3). The mean age and follow-up were 12.3 years (range, 3 to 17 years) and 12 months (range, 2 to 44 months), respectively. There were 10 males. Chemotherapy (n = 6), idiopathic, trauma, and gallstone-pancreatitis (n = 2 each) accounted for the aetiology in the majority of the cases. (Table 1).

### 3.2. PFC Characteristics

A LAMS was inserted for symptomatic PFC (n = 2), WON (n = 7), pseudocysts (n = 4), and as a rescue for recurrent pseudocysts after initial endoscopic ultrasound-guided aspiration (n = 3) or percutaneous drainage (n = 1) or laparoscopic debridement (n = 1). The interval from presentation to the insertion of the LAMS was described in three cases (1, 4, and 12 weeks, respectively) [20,21,22]. The size of the PFC at the LAMS insertion ranged from 5.5 to 22 cm. Two sizes of the LAMS were described, with 5 children using 10 × 10 mm LAMSs, and 13 children using 10 × 15 mm LAMSs, including the youngest child who was 3 years of age at the time of the LAMS insertion. The choice of LAMS size was not clearly dictated by the size or type of the PFC. Of the 18 children, 7 had one, and 4 had multiple endoscopic necrosectomies performed via the LAMS to achieve resolution of PFCs. For children requiring multiple endoscopic necrosectomies, the interval between necrosectomies was reported in three children and ranged from 4 to 14 days. The mean duration for which the LAMS was kept in situ was 41 days (range, 7 to 100 days) (Table 2).

**Table 2:** LAMS details and treatment outcomes from systematic review.

LAMS Details and Treatment (N = 18)	n (%)
Stent size (mm)	
10 × 15 mm	13 (72%)
10 × 10 mm	5 (28%)
Time to resolution, days, mean (range)	40 (7–100)
Pseudocyst	41.6 (19–100)
Walled-off necrosis	40.6 (28–56)
Stent indwelling duration, days, mean (range)	40 (7–100)
No. of cases requiring necrosectomy, n (%)	8 (44%)
Interval between necrosectomies, days, mean (range)	7 (4–14)
Follow-up duration, months, mean (range)	12 (2–44)

### 3.3. Outcomes

All studies reported 100% technical success with no procedural complications. All children recovered with resolution of their PFCs and without any recurrence over the duration of follow-up (12 months, range 2–44 months). Endoscopic removal of LAMSs was uneventful in all children. Migration of LAMSs into the colon (following collapse of the WON) was reported in one case (Table 3).

**Table 3:** Studies on paediatric pancreatic fluid collections (PFCs) drainage with lumen-apposing metal stents (LAMSs) 2015–2024.

Author/Year	No.	Age (Year)	M/F	Aetiology	Type of PFC	Indication for Drainage	Time to LAMS Insertion (Days)	PFC Size (cm)	Stent Size (mm)	Time to Resolution (Days)	LAMS Indwell Duration (Days)	No. of Necro-sectomy	Necrosectomy Interval (Days)	Follow-Up (Months)
Nieto et al., 2015 [19]	3	3–16	3 M	2 Trauma 1 Alcohol	1 pseudocyst 2 WON (3 first-line)	Unknown	Unknown	5.5–14	10 × 15	30	30	1 1 0	Unknown	2–9
	1	7	F	Familial	Pseudocyst (Rescue, EUS aspiration)	Pressure	Unknown (third recurrence)	7	10 × 10	30	30	0	NA	4
	1	6	F	Metastatic neuro- blastoma	Pseudocyst (Rescue, EUS aspiration)	Pressure	Unknown (second recurrence)	7.6	10 × 10	30	30	0	NA	4
Trindade et al., 2016 [21]	1	14	M	Psychiatric Medication	WON (First-line)	Sepsis	Unknown	10	10 × 10	56	56	1	7	2
Giefer et al., 2016 [22]	1	11	F	Gallstone	Symptomatic PFC (First-line)	Pressure	28	20	10 × 15	30	14	3	Unknown	2
Bang et al., 2016 [23]	1	17	F	Idiopathic	WON (First-line)	Unknown	Unknown	6	10 × 15	Un-known	56	0	NA	26 (median)
Kim C et al., 2019 [24]	2	17 12	M M	Gallstone Idiopathic	Pseudocyst WON	Abdominal pain Abdominal pain	Unknown Unknown	11 10	10 × 10 10 × 15	21 Unknown	21 Unknown	0 2	NA Unknown	Unknown
Costa et al., 2020 [25]	1	12	F	Chemo- therapy	Pseudocyst (First-line)	Pressure	Unknown	Unknown	10 × 15	100	100	0	NA	Unknown
	1	17	M	Chemo- therapy	Pseudocyst (Rescue, percutaneous drainage)	Pressure	Unknown (First recurrence)	Unknown	10 × 15	34	34	0	NA	Unknown
	1	11	M	Chemo- therapy	Pseudocyst (First-line)	Pressure	Unknown	Unknown	10 × 15	82	82	2 (D37, D41)	4	Unknown
Li et al., 2023 [26]	1	15	F	COVID-19	WON (First-line)	Sepsis	Unknown	9	10 × 15	42	42	4	7–14	18
Fiumana et al., 2023 [27]	1	11	F	Chemo- therapy	Symptomatic PFC (First-line)	Pressure	19	13	10 × 10	7	7	1	NA	8
Pasqualetto et al., 2024 [20]	1	15	F	Pancreatic leak post surgery	Pseudocyst (Rescue, laparoscopic debridement)	Pressure	7	10	10 × 15	19	21	0	NA	44
	1	15	M	Chemo- therapy (B ALL)	Pseudocyst (Rescue, EUS aspiration)	Sepsis (fever)	Unknown (second recurrence)	12	10 × 15	28	28	0	NA	15
	1	10	M	Chemo- therapy (T LL)	WON (First-line)	Pressure	Unknown	22	10 × 15	48	48	0	NA	12
Our experience	1	10	M	Steroids	WON (First-line)	Sepsis	21	19.1	10 × 15	28	28	3	7	7
	1	17	M	PEG-asparaginase	WON (First-line)	Pressure	49	12.2	10 × 15	50	50	2	50	1

## 4. Discussion

We report our initial experience with two cases and the evidence from our literature review on endoscopic trans-gastric drainage of PFCs using LAMSs in paediatric acute pancreatitis. In our limited experience with two cases performed by a senior adult interventional endoscopist with paediatric expertise, LAMSs were indicated for PFCs causing extrinsic pressure effects and/or sepsis. In expert hands, LAMS insertion was a technical success and achieved drainage of the PFCs. There were no complications related to the insertion or during the stent dwell period of 28 and 50 days, respectively.

Furthermore, we narrate the synthesised evidence from a review of 18 children who underwent endoscopic transgastric LAMS insertion for the drainage of PFCs due to acute pancreatitis from nine studies published between 2015 and 2024, with the initial paediatric report in the literature in 2015 [10,19].

The endoscopic management of PFCs in acute pancreatitis evolved from plastic stents to FCSEMS and, recently, to the use of LAMSs.The most common indications for LAMSs in paediatric pancreatitis include first-line treatment for WON and drainage of recurrent pseudocyst after prior intervention [18,21,23,24,25,26,27,28].

EUS-guided transgastric LAMSs achieved 100 % technical and clinical success in all children. The prerequisites for the technical success of FCSEMS in children were the maturity of the PFCs to 4 weeks or longer, proximity to the stomach wall (<1.5 cm), and a body weight in excess of 15 kg [1]. Similarly, the criteria for LAMS success in children were deemed to be (a) maturity of the PFCs with a well-defined wall thicker than 1 mm, and (b) a < 1 cm gap between the adjacent walls of the PFCs and the stomach, with the body weight not finding mention as a criterion in our systematic review [20]. NASPGHAN recommends a minimum interval of 4 weeks to intervention for necrosis in paediatric pancreatitis [5]. The interval between initial presentation and insertion of LAMSs in our systematic review ranged from 1 to 19 weeks, with the shortest interval of 1 week being for postoperative PFCs following pancreatectomy, which recurred despite laparoscopic drainage. Pre-LAMS Magnetic Resonance Imaging had characterised the wall of the PFC, which was abutting the stomach [20]. In our first case, LAMS insertion at 3 weeks was a technical and clinical success because of progressive thickening of the wall of the PFC, which was adjacent to the stomach. Size is a criterion in adult guidelines for LAMS drainage of PFCs [23]. However, based on our review, size or age alone may not influence the success of LAMSs since the youngest child was 3 years old and the largest PFC was 22 cm in size [19,20].

As a rescue measure, LAMS insertion was successful for drainage of PFCs in five children following either endoscopic ultrasound-guided aspiration (n = 3), percutaneous drainage (n = 1), or laparoscopic drainage (n = 1) [19,20,25].

The excellent success of LAMSs may be due to its unique configuration, which enables ease of technical insertion by a single operator with reduced exchanges. EUS maps a passage between the stomach wall and the PFC, thereby obviating the need for either a visible bulge in the gastric lumen or a guidewire or fluoroscopy as a guide to the PFC, and ensures Doppler identification and avoidance of any sizeable blood vessels during deployment. The electrocautery-based catheter delivery system reduces the risk of intra-procedural bleeding [23]. The saddle shape with the flanges at the two ends ensures close apposition between the walls of the PFC and the stomach, thereby preventing dislocation or migration. Reportedly, the LAMS is amenable to endoscopic repositioning in case of mis-deployment at the initial procedure, which was not required in any of our cases or in this systematic review [29]. The effective lumen obviates the need for dilatation, ensures efficient drainage and provides access for endoscopic necrosectomy through the stent. Direct endoscopic necrosectomy, either at the time of insertion of the LAMS or subsequently, was indicated for clinically symptomatic collections with debris. Although it is conceivable that food particles may enter and occlude the LAMS, this has not been observed in our two cases and in this systematic review. Therefore, patency measures such as multiple pigtail stents within the lumen of the LAMS are not necessary.

Paediatric guidelines recommend LAMS removal within 4 weeks for pseudocysts and 6 weeks for WON [2]. Our literature review revealed that the mean dwell time for LAMS was up to 6 weeks (41 days). Five children tolerated a stent in dwell time beyond 6 weeks without any complications over a mean follow-up of 13.3 months (range, 2–26 months). The reported maximum duration of the stent dwell was 100 days for persistence of pseudocyst due to a disrupted pancreatic duct that required a pancreatic stent 1 month after LAMS insertion [25]. LAMS removal could be performed with basic endoscopic techniques. Our review and institutional experience demonstrated no recurrence after LAMS removal without insertion of prophylactic plastic stents at LAMS removal. Therefore, the LAMS was effective in reducing the need for additional interventions for PFC in children, which agrees with published literature in the adult population [28].

Multi-centre adult studies report the risk of perforation (5%) and bleeding (14%) with direct endoscopic necrosectomy, and stent burial with prolonged indwelling from tissue in-growth, pseudoaneurysm, and bleeding from mucosal erosion [30,31,32]. These were not observed in our two cases, and in this systematic review which included a maximum stent dwell of 50 days and 100 days, respectively. We speculate that increasing application of LAMS may further reveal its safety profile in the paediatric population in comparison to published large-scale adult studies [33].

Our study is limited by short follow-up time (2–44 months). In addition, the literature on this emerging field in children consists largely of case reports and small case series. Due to the limited numbers, comparisons with plastic stents and adult literature, including meta-analyses, were not performed because of the possible skewed data for paediatric LAMSs.

To the best of our knowledge, guidelines for endoscopic LAMSs for PFCs, especially WON, in paediatric pancreatitis are forthcoming. Further to the initial success with our first two cases, we are inclined to cautiously adopt endoscopic transgastric LAMSs as our initial approach for the drainage of WON in paediatric pancreatitis, as guided by CT characterisation of maturity and proximity to the stomach.

## 5. Conclusions

In conclusion, endoscopic transgastric LAMSs, together with endoscopic necrosectomy when indicated, may be a technically feasible method of drainage of WON in acute pancreatitis in children with minimal complications. Centres with expertise in advanced therapeutic endoscopy may consider LAMSs as the first-line in a step-up approach for the drainage of pancreatic WON in conjunction with a multidisciplinary team for the management of complicated pancreatitis in children. Large, multi-centre paediatric studies may further characterise the efficacy and safety of LAMSs in comparison to other stents and drainage procedures in the adult population.

## Figures and Tables

**Table 1:** Patient characteristics from systematic review.

Patient Characteristics (N = 18)	n (%)
Mean Age, years, mean (range)	12.3 (3–17)
No. of males, n (%)	10 (55%)
Aetiology of pancreatitis, n (%)	
Chemotherapy	6 (33%)
Trauma	2 (11%)
Idiopathic	2 (11%)
Gallstone pancreatitis	2 (11%)
Others	6 (33%)
Types of PFC, n (%)	
Symptomatic PFC	2 (11%)
Pseudocyst	9 (50%)
WON	7 (39%)
Size of PFC, cm, mean (range)	11.2 (5.5–22)
Indication of LAMS, n	
Pressure effect of PFC	9
Persistent/recurrent abdominal pain	2
Sepsis	3
Not stated	4
First-line treatment ^1^, n	12 (66%)
Rescue treatment ^1^, n	
Post EUS aspiration	3
Post percutaneous drainage	2
Post laparoscopic debridement	1
Time from presentation of PFCs to LAMS insertion, weeks, mean (range)	8 (1–19)

^1^ LAMS can be deployed as first-line treatment or as rescue after failure of other drainage modalities.

## Data Availability

Data sharing is not applicable as no separate datasets were generated and/or analysed for this study.

## Supplementary Materials

The following supporting information can be downloaded at https://www.mdpi.com/article/10.3390/children12080965/s1, Evaluation of studies using JBI.

## Abbreviations

The following abbreviations are used in this manuscript:

PFC	Pancreatic fluid collections
WON	Walled-off necrosis
LAMS	Lumen-apposing metal stent
NUH	National University Hospital
NASPGHAN	North American Society for Paediatric Gastroenterology, Hepatology and Nutrition
FCSEMS	Biliary fully covered self-expanding metallic stents
CT	Computerised tomography scan
EUS	Endoscopic ultrasound
INR	International Normalized Ratio (coagulation)
