# Supplementary material for: Lumen-Apposing Metal Stents for Endoscopic Transgastric Drainage of Pancreatic Fluid Collections in Children—A Case Report and Review of Safety and Efficacy
Source: Children (Basel). 2025 Jul 23;12(8):965. doi: 10.3390/children12080965 (PMC12384561; doi:10.3390/children12080965)
Supplement: Supplementary file 1 [file children-12-00965-s001.zip › children-3677173-supplementary.pdf]

Evaluation of studies using JBI

Author/ Year	Nieto et al. 2015 [18]	Trindade et al. 2016 [21]	Giefer et al. 2016 [22]	Bang et al. 2016 [25]	Costa et al. 2020 [26]	Li et al. 2023 [27]	Fiumana et al. 2023 [28]	Pasqualetto et al.2024 [20]	Kim et al. 2019 [23]
Review question	Y	Y	Y	Y	Y	Y	Y	Y	Y
Inclusion criteria	Y	Y	Y	Y	Y	Y	Y	Y	Y
Search strategy	NA	NA	NA	NA	NA	NA	Y	NA	NA
Sources and resources used for search adequate?	NA	NA	NA	NA	NA	NA	Y	NA	NA
Criteria for appraising studies appropriate?	NA	NA	NA	NA	NA	NA	Y	NA	NA
Critical appraisal conducted by 2 or more reviewers independently?	NA	NA	NA	NA	NA	NA	Y	NA	NA
Any method to minimize errors in data extraction?	Unclear*	N	Unclear*	Y	N	N	N	Y	N
Methods to combine studies appropriate?	Y	NA	Y	Y	Y	NA	Y	Y	Y
Likelihood of publication bias?	NA	NA	NA	NA	NA	NA	N	N	N
Recommendations supported by reported data?	Y	Y	Y	Y	Y	Y	Y	Y	Y
Specific directives for new research appropriate?	NA	Y	Y	Y	Y	NA	Y	Y	Y
Include/exclude	Include	Include	Include	Include	Include	Include	Include	Include	Include
